# Assessing the Diversity and Biomedical Potential of Microbes Associated With the Neptune’s Cup Sponge, *Cliona patera*

**DOI:** 10.3389/fmicb.2021.631445

**Published:** 2021-06-29

**Authors:** Xin Yi Ho, Nursheena Parveen Katermeran, Lindsey Kane Deignan, Ma Yadanar Phyo, Ji Fa Marshall Ong, Jun Xian Goh, Juat Ying Ng, Karenne Tun, Lik Tong Tan

**Affiliations:** ^1^Singapore Centre for Environmental Life Sciences Engineering, Nanyang Technological University, Singapore, Singapore; ^2^Natural Sciences and Science Education, National Institute of Education, Nanyang Technological University, Singapore, Singapore; ^3^National Parks Board, Singapore Botanic Gardens, Singapore, Singapore

**Keywords:** bacterial diversity, quorum-sensing inhibition, MS/MS-molecular networking, amplicon sequencing, metabolomics

## Abstract

Marine sponges are known to host a complex microbial consortium that is essential to the health and resilience of these benthic invertebrates. These sponge-associated microbes are also an important source of therapeutic agents. The Neptune’s Cup sponge, *Cliona patera*, once believed to be extinct, was rediscovered off the southern coast of Singapore in 2011. The chance discovery of this sponge presented an opportunity to characterize the prokaryotic community of *C. patera*. Sponge tissue samples were collected from the inner cup, outer cup and stem of *C. patera* for 16S rRNA amplicon sequencing. *C. patera* hosted 5,222 distinct OTUs, spanning 26 bacterial phyla, and 74 bacterial classes. The bacterial phylum *Proteobacteria*, particularly classes *Gammaproteobacteria* and *Alphaproteobacteria*, dominated the sponge microbiome. Interestingly, the prokaryotic community structure differed significantly between the cup and stem of *C. patera*, suggesting that within *C. patera* there are distinct microenvironments. Moreover, the cup of *C. patera* had lower diversity and evenness as compared to the stem. Quorum sensing inhibitory (QSI) activities of selected sponge-associated marine bacteria were evaluated and their organic extracts profiled using the MS-based molecular networking platform. Of the 110 distinct marine bacterial strains isolated from sponge samples using culture-dependent methods, about 30% showed quorum sensing inhibitory activity. Preliminary identification of selected QSI active bacterial strains revealed that they belong mostly to classes *Alphaproteobacteria* and *Bacilli*. Annotation of the MS/MS molecular networkings of these QSI active organic extracts revealed diverse classes of natural products, including aromatic polyketides, siderophores, pyrrolidine derivatives, indole alkaloids, diketopiperazines, and pyrone derivatives. Moreover, potential novel compounds were detected in several strains as revealed by unique molecular families present in the molecular networks. Further research is required to determine the temporal stability of the microbiome of the host sponge, as well as mining of associated bacteria for novel QS inhibitors.

## Introduction

Marine sponges are filter feeders and important constituents of benthic environments, with diversity often exceeding that of corals and algae (Bell, 2008). In addition to providing habitat for a wide range of reef fauna, such as fishes and other invertebrates (Bell, 2008), sponges are known to host highly specific and dense microbial communities, which can comprise up to 40% of the sponge biomass (Vacelet and Donadey, 1977; Hentschel et al., 2003, 2006; Webster and Taylor, 2012). As such, the sponge-microbial composition is considered to be one of the most complex and diverse holobionts in the marine habitat. Studies found that the most dominant bacterial symbiont groups are from the phyla *Proteobacteria* (mainly *Gamma*- and *Alphaproteobacteria*), *Actinobacteria*, *Chloroflexi*, *Nitrospirae*, *Cyanobacteria* and candidatus phylum *Poribacteria*, while *Crenarchaeota* represents the major archaeal group (Thomas et al., 2016). Based on the abundance of these microbial communities, sponges are broadly classified into two groups, namely high microbial abundance (HMA) sponges and low microbial abundance (LMA) sponges (Hentschel et al., 2003, 2006; Moitinho-Silva et al., 2017). Moreover, the composition of the symbiotic microbial community within sponges is generally host specific and such associations appear to be relatively stable temporally, over different geographical locations, and under various environmental conditions (Pita et al., 2013; Turon et al., 2019).

The sponge microbiome is known to provide a number of valuable contributions to many aspects of the host physiology and ecology as well as mediation of nutrient cycles, such as carbon, nitrogen, phosphorus, and sulfur cycles (Webster and Thomas, 2016; Zhang et al., 2019). Due to the challenges associated with culturing microbial symbionts from sponges, culture-independent approaches, including metabarcoding, metagenomic, metaproteomic, metabolomic and metatranscriptomic techniques, have been instrumental in obtaining information to infer the putative functional roles of these sponge symbionts (Thomas et al., 2010; Fan et al., 2012; Liu et al., 2012; Radax et al., 2012; Gauthier et al., 2016; Mohanty et al., 2020). Metagenomic differences between sponge-associated and seawater microbial consortia highlighted genes enriched only in sponge symbionts that are relevant to the symbiosis (Fan et al., 2012; Hentschel et al., 2012; Horn et al., 2016). For example, genomic and metagenomics studies, including metagenomic-assembled genomic approach, have shown that the sponge symbionts are enriched in genes related to the synthesis of vitamins, such as vitamins B_1_, B_2_, B_7_, and B_12_, as well as enzymes involved in various metabolic and biosynthetic pathways (Thomas et al., 2010; Fan et al., 2012; Fiore et al., 2015; Lackner et al., 2017; Bayer et al., 2020; Engelberts et al., 2020; Podell et al., 2020; Storey et al., 2020; Robbins et al., 2021). In addition, microbial symbionts have been revealed to be a source of secondary metabolites, such as polyketides and peptides, which are responsible for conferring chemical defense of the sponge holobiont (Paul et al., 2019). Having a diverse community of microbial symbionts can expand the metabolic capabilities of the sponge hosts and provide functional redundancy of specific metabolic pathways (Ribes et al., 2016). For instance, sponges are known to produce large amounts of ammonia as a metabolic waste product. As such, it is not surprising that sponge symbionts are found to be rich in genes related to nitrogen metabolism, especially ammonia oxidation as well as initial steps of denitrification (e.g., nitrate and nitrite reduction) (Siegl et al., 2011; Liu et al., 2012; Bayer et al., 2014; Moitinho-Silva et al., 2014; Li et al., 2016). Certain sponge species benefit from harboring dense populations of photosynthetic *Cyanobacteria* through the translocation of photosynthates, mostly in the form of glycerol, from the symbionts to the host (Wilkinson, 1987; Freeman and Thacker, 2011). Moreover, studies have shown that some sponges derive up to 50% of their energy needs and 80% of carbon budget from symbiotic *Cyanobacteria* (Wilkinson, 1983; Cheshire and Wilkinson, 1991).

Since the early 1970s, marine sponges have been known to be a rich source of novel natural products with unprecedented chemical scaffolds as well as potent biological activities (Sipkema et al., 2005; Mehbub et al., 2014). As such, these ancient metazoans are able to provide potential therapeutic agents against a myriad of diseases, ranging from cancer, infectious diseases, inflammation to malaria (Hentschel et al., 2012; Andersen, 2017). The chemical ecology of sponge natural products has been investigated for decades and it is known that these compounds serve as chemical defenses against predators, microorganisms, fouling organisms, and other competitors (Puglisi et al., 2019; Paul et al., 2019). There is increasing evidence that there is significant overlap between the biosynthetic machinery of marine bacteria with that of chemically prolific sponges. It is now known that many compounds that were originally reported from sponges are produced by the host microbiome (Haygood et al., 1999; McCauley et al., 2020). For instance, the recently identified symbiotic “Entotheonella” bacteria of the phylum Entotheonellaeota were identified as the key producers of bioactive metabolites, such as polyketides and peptides, found in the lithistid sponge *Theonella swinhoei* (Wilson et al., 2014). In another recent study, a multiproducer microbial consortium was found to be the basis of chemical diversity of the New Zealand sponge, *Mycale hentscheli* (Rust et al., 2020; Storey et al., 2020). The symbiotic cyanobacterium, *Oscillatoria spongeliae*, has also been identified as a source of halogenated natural products in dysideid sponges, such as *Lamellodysidea herbacea* (Unson et al., 1994; Flatt et al., 2005; Agarwal et al., 2017). The realization of symbiotic bacteria as the true biogenetic sources of bioactive natural products in sponges has led to the use of innovative technology and “omics” approaches, including metagenomics and metabolomics, to unlock the biomedical potential of these microbes for the production of potential drug agents (Steinert et al., 2014; Helber et al., 2019; Ong et al., 2019; Paul et al., 2019).

In the current study, both culture-independent and culture-dependent methods are used to assess the diversity and biomedical potential of marine bacteria associated with the Neptune’s Cup sponge, *Cliona patera* from Singapore. Arguably one of the most iconic and famous sponge species in the world, *Cliona patera* (as *Spongia patera*) was the first sponge species to be described from Singapore (Hardwicke, 1822; Low, 2012). In nature, it is usually shaped like a wine glass and can grow to over a meter in height and diameter. *C. patera* was common and abundant in Singapore waters in the early 19th century. However, due to its impressive size, it was much sought after by natural history museums and private collectors in the past. Since the early 20th century, this sponge has not been recorded from this region, and was thought to be extinct (Lim et al., 2012). In early 2011, during a routine survey dive, this sponge was fortuitously rediscovered off Singapore’s southern islands (Lim et al., 2012). Another population of *C. patera* was recently discovered near the islands of Koh Rong Sanloem and Koh Koun, Cambodia in 2018. The reappearance of *C. patera* in Singapore waters presents a unique opportunity to study the sponge in its natural habitat, including the diverse associated microbes. This study aimed to evaluate the diversity of the *C. patera* associated prokaryotic community found on the top oval concave disk and stalk of the gamma stage (massive, free-living) of the sponge using 16S rRNA amplicon sequencing (Lim et al., 2012; Zhang et al., 2016). In addition, sponge-associated bacteria obtained from two sponges were cultured and their extracts assessed for quorum sensing inhibitory (QSI) activity based on a *Pseudomonas aeruginosa* PAO1 *lasB-gfp* biosensor strain. The disruption of bacterial quorum sensing systems in pathogenic bacteria using small molecules represents a novel chemotherapy against infectious diseases (Saurav et al., 2016). Furthermore, mass spectroscopic-based metabolomics method, using the Global Natural Product Social Molecular Networking platform, was used to annotate the metabolomes of selected QSI active marine bacterial strains (Nguyen et al., 2013).

## Materials and Methods

### Collection of *Cliona patera* Samples

Sponge samples from *Cliona patera* (Class Demospongiae; Family Clionaidae; gamma stage) were collected at two time points, approximately 1.5 months apart on January 14, 2019 and February 27, 2019, in the waters surrounding the southern coast of Singapore along the Singapore Strait at depth of about 12 m (National Parks Board Permit Number: NP/RP19-036) (Lim et al., 2012). In the first collection, a total of 12 sponge samples, each measuring approximately 3 cm^1^, from two *C. patera* colonies, designated as NP1 and NP6, were obtained. A second collection of 36 sponge samples from six colonies of *C. patera*, including NP1 to NP6, was carried out. The height of the sponges ranged from 47 to 61 cm (Supplementary Figure 1). From each sponge, a total of six samples were excised: two samples from the inner surface of the sponge cup (Inner), two samples from outer surface of the sponge cup (Outer), and two samples from stem of sponge (Stem). Upon collection, sponge samples were placed individually in Nasco^TM^ Whirl-Pak^®^ bags containing seawater. Samples were divided equally for microbial analysis using culture-dependent and culture-independent methods (see below sections). Samples reserved for microbial diversity study, using culture-independent method, were drained of seawater and immediately frozen in a dry shipper, then stored at −80°C until time of processing. Samples for culture-dependent methods were transported to the laboratory for immediate processing within 2.5 h of sample collection.

In addition to sponge samples, seawater (two and three samples in January 2019 and February 2019, respectively) was collected in 1 L-polypropylene Nalgene^®^ bottles (previously sterilized by autoclaving at 121°C for 15 min) and placed on ice in a cooler for transportation to the Singapore Centre for Environmental Life Sciences Engineering (SCELSE), Nanyang Technological University (NTU). In the laboratory, each seawater sample was filtered through a 0.2 μM cellulose acetate filter paper (Sartorius Stedim), which was stored at −80°C until time of processing.

### Microbial DNA Extraction and 16S rRNA Analysis

DNA was extracted from each sponge sample and seawater filter using the DNeasy^®^ PowerSoil Kit (QIAGEN) according to the manufacturer’s instructions. Concentration and purity of the DNA were determined using a Nanodrop^TM^ 2000 spectrophotometer (Thermo Fisher Scientific) and Qubit^TM^ 2.0 fluorometer (Thermo Fisher Scientific). To study the microbial communities in the sponge samples, PCR was used to amplify the V4 region of the 16S rRNA gene using the 515F and 806R primers (Caporaso et al., 2011). PCR reactions required 12.5 μL of KAPA HiFi HotStart ReadyMix 2X, 5 μL of primers (1 μM) and 2.5 μL of 5 ng/μL of DNA template. The mixture was adjusted to a final volume of 25 μL using sterile water. The reaction was cycled in a thermocycler for an initial denaturation at 95°C for 3 min, denaturation at 98°C, annealing at 52°C and extension at 72°C (30 s, 30 cycles) and a final extension at 72°C for 5 min. Gel extraction was employed to excise the band of interest from the pooled PCR products using the Invitrogen PureLink^TM^ Quick Gel Extraction Kit (Thermo Fisher Scientific) according to manufacturer’s instructions. The quality of the purified PCR products was checked using the Agilent 2200 Tape station before they were sent to sequencing facility at SCELSE, NTU for amplicon sequencing on the Illumina MiSeq Platform.

Raw sequencing data were processed in Mothur v 1.31.1 (Kozich et al., 2013) using the MiSeq SOP pipeline^2^. Sequence reads of low quality were removed and the remaining sequences trimmed and aligned (maxambig = 0, minlength = 309, maxlength = 379, maxhomop = 8). Pre-clustering was done on sequences using the Deblur method (Amir et al., 2017) and chimeras were removed with VSEARCH. Taxonomy was assigned with the SILVA reference database version 132 to identify the sub-Operational Taxonomic Units (OTUs) following the application of Deblur. Non-bacterial and non-archaeal (i.e., mitochondria, chloroplast, eukaryotes, and unclassified) reads were filtered out. All sequencing data were uploaded to the NCBI Sequence Read Archive under BioProject accession number PRJNA623254.

### Statistical Analysis

For each sample group, mean relative abundance of the representative taxa was calculated. Using Mothur, the data were rarefied to 55,140 sequence reads per sample (Weiss et al., 2017). Alpha diversity was assessed in terms of S_*obs*_ (observed richness; number of OTUs), Chao (estimated richness), Shannon evenness and Inverse Simpson Diversity. To test for difference in means amongst sample groups, Analysis of variance (ANOVA) was conducted in R v 3.6.1 with *post hoc* Tukey tests used to identify significantly different groups. Beta diversity, or prokaryotic community structure, among groups was examined in PRIMER v 7 using permutational multivariate analysis of variance (PERMANOVA) with Bray-Curtis similarity matrices of square root transformed data and visualized in non-metric multidimensional scaling (nMDS) plots. Additionally, permutational multivariate analysis of dispersion (PERMDISP) was used to test for homogeneity of dispersion among samples within groups. The specific OTUs contributing to the differences observed in the PERMANOVA analysis were determined by running multivariate generalized linear models (GLMs) with a negative binomial distribution. The GLMs were performed in R v 3.6.1 using the mvabund package (Wang et al., 2012) with data subsampled to the top 500 OTUs to focus the analysis on the most abundant OTUs present in the sponge samples. OTUs of interest were screened using the nucleotide-nucleotide BLAST search to determine closely related sequences (Altschul et al., 1990).

### Cultivable Marine Bacterial Isolation From Sponge Samples

Sponge samples from the inner cup, outer cup and stem were washed with sterile artificial sterile seawater to remove loosely attached microorganisms. They were then homogenized thoroughly with a mortar and pestle with 10 mL of sterile artificial seawater for the purpose of bacterial isolation. Fifteen different marine media, including A1 to A5, marine agar, starch casein agar, actinomycete isolation agar, YEME, YPG, A1 + C, YMP, YMP + C, TCG, and SIM were used for the isolation of cultivable marine bacteria from the first sponge collection (Supplementary Table 1). All media, containing Difco^TM^ Marine agar, were sterilized by autoclaving before supplemented with concentration of potassium dichromate/cycloheximide and nalidixic acid sodium salt solution to minimize fungal growth and fast growing Gram-negative bacteria, respectively. The antibiotic solution was filtered (0.2 μm pore size) before being added to the autoclaved media in petri dishes as isolation plates. The homogenized supernatants of sponge samples were heat-shocked at 65°C for 10 min to reduce the growth of fast growing Gram negative bacteria. Twenty microliters of the heat-shocked supernatant was then inoculated in duplicates onto isolation agar plates and incubated at 25°C for 6–8 weeks.

All isolation agar plates were checked every day and the bacterial isolates were picked or selected based on the criteria of colonies having unique or interesting morphology, including matte textures, irregular colony shapes, and bright colors. They were then re-streaked onto MBA medium (Difco^TM^ Marine Agar 2216) and incubated at 25°C for approximately 3–7 days. The isolated marine bacterial strains were then inoculated into 50 mL falcon tubes containing 20 mL of marine broth (Difco^TM^ Marine Broth 2216), supplemented with nalidixic acid sodium salt and potassium dichromate and incubated at 25°C with 150 rpm shaking for 14 days and stored at 5°C while awaiting further processing.

### Small-Scale Fermentation of Marine Bacterial Strains and Preparation of Organic Extracts

Ten milliliters of each isolated marine bacterial strain, maintained in marine broth, was transferred into a 250 mL-Erlenmeyer flask containing 100 mL of marine broth supplemented with nalidixic acid sodium salt and potassium dichromate and incubated at 25°C with 150 rpm shaking for 14 days. After the incubation period, an equal volume of ethyl acetate (EtOAc) was added to the liquid cultures and the mixture were transferred into separatory funnel to collect the EtOAc phase. Solvent partitioning using EtOAc was repeated twice for each liquid culture. EtOAc was removed *in vacuo* using a rotary evaporator, and the dried extracts were reconstituted with equal parts absolute ethanol and isopropanol before transferring to scintillation vials, stored at −20°C, for subsequent biological evaluation in the QSI assay and metabolomics analysis.

### *Pseudomonas aeruginosa* Quorum Sensing Inhibitory Assay

The bacterial quorum sensing inhibitory (QSI) assay was conducted based on the biosensor strain, *Pseudomonas aeruginosa* PAO1 *lasB-gfp*(ASV) (Hentzer et al., 2002). The monitor strain has their promoter fused to an unstable GFP (green fluorescent protein) that has a C-terminal oligopeptide extension containing the amino acids ASV [*gfp*(ASV)]. This causes the GFP to be more susceptible to degradation by housekeeping proteases, resulting in a short half-life. As such, unstable *gfp*(ASV) allows for monitoring of temporal QS-regulated gene expression (Andersen et al., 1998). The GFP, composed of 238 amino acid residues (26.9 kDa), exhibits bright green fluorescence when exposed to light in the blue to ultraviolet range. The *lasB* gene is responsible for the production of the virulence factor, elastase, which can cause degradation of elastin, collagen as well as necrosis of macrophages in host during infections (Lee and Zhang, 2015). Marine bacterial-derived extracts were tested in triplicates and prepared in a 96-well microtiter plates. Stock solutions of extracts were prepared in 100% DMSO at concentration of 1 mg/mL. Each bacterial stock solution was then mixed with ABTGC medium (Supplementary Table 1) and serial diluted to give a final concentration of 200 μg/mL in each well of the microtiter plate. A culture of the PAO1 *lasB*-*gfp* strain was grown overnight in Luria-Bertani medium at 37°C with 200 rpm shaking. The culture was subsequently diluted with ABTGC medium to a final optical density at 600 nm (OD_600_) of 0.02. An equal amount of the bacterial suspension was added to the wells to reach final inhibitor concentration of 100 μg/mL. All bacterial extracts were initially screened at a concentration of 100 μg/mL before proceeding with dose-dependent assay (ranging from 100 to 1.563 μg/mL using ABTGC medium for dilution) for selected extracts that showed significant QSI activity. DMSO control (0.1% final concentration) and blank control were added into the microtiter plates in triplicates. The microtiter plates were then incubated at 37°C for 17 h in a Tecan Infinite 200 Pro plate reader (Tecan Group Ltd., Maännedorf, Switzerland) to measure fluorescence and cell density. Bacterial growth was measured at an optical density at 450 nm while fluorescence was measured as excitation and emission wavelength at 485 nm and 535 nm, respectively. Expression of GFP was normalized by dividing its value with the growth measured at the respective time points over a period of 10–16 h. Bacterial extracts were further tested using PAO1-*gfp* strain to ensure that they were targeting the QS genes and not the GFP (Yang et al., 2007). Bacterial extracts that did not interfere with fluorescent output signals in the PAO1-*gfp* strain were then subjected to dose-dependent QS inhibition study.

### Mass Spectrometry-Based Molecular Network of QSI Active Marine Bacterial Extracts

Quorum sensing inhibitory active marine bacterial extracts were filtered over C_18_ SPE cartridges by application of 1.0 mL sample (1 mg/mL) and elution with 3 mL acetonitrile (CH_3_CN). Solvent was removed *in vacuo* using a rotary evaporator before being redissolved in 1 mL CH_3_CN, then vortex mixing over 5 min and transferred into separate Eppendorf tubes. Tubes were then centrifuged at 10,000 rpm at 4°C over 10 min and the supernatant was aliquoted and diluted with CH_3_CN to 10,000 × dilution. One-and-a-half microliters of each diluted sample was subjected to LC-HRMS/MS [Q Exactive Plus Hybrid Quadrupole-Orbitrap Mass Spectrometer (Thermo Fisher Scientific) equipped with a heated electrospray ionization (H-ESI) probe] performed with a Thermo Scientific Hypersil GOLD (C_18_ 50 mm × 2.1 mm, 1.9 μm) column and maintained at a column temperature of 40°C and sample temperature of 4°C using a gradient elution program of 0.1% aq. HCOOH (mobile phase A) and 98% CH_3_CN in 0.1% aq. HCOOH (mobile phase B) at a flow rate of 0.5 mL/min. The gradient program began at 10% and increased to 50% of mobile phase B within 2 min and was held at 50% mobile phase B for 2 min. It was then increased to 100% of mobile phase B within 6 min and was held at 100% B for 0.5 min before reconditioning back to the starting composition in 0.5 min and was held at the starting composition of 10% B for 3 min bringing the total run time to 14 min. All the mass spectra were collected in the positive ion and data-dependent acquisition mode, where the first five most intense ions of each full-scan mass spectrum (mass range: *m/z* 100–1500) were subjected to tandem mass spectrometry (MS/MS) analysis. MS scan time of 0.25 s over 14 min, MS/MS scan time of 0.06 s, and a 3-step normalized collision energy of 25, 35, and 55. The MS/MS data files were converted from .*RAW* to .*mzXML* files using MSConvert software and uploaded to the Global Natural Product Social Molecular Networking (GNPS) server^3^ and the molecular networking performed using the GNPS data analysis workflow employing a special spectral clustering algorithm.

The network spectra and the library reference spectra were required to have a minimum cosine score threshold of 0.7 and a minimum of six matched peaks in order to be considered for spectral library annotation and a minimum of six matched fragment ions. The precursor ion mass tolerance was set at 2.0 Da and a MS/MS fragment ion tolerance of 0.5 Da to create a consensus spectra. Further edges between two nodes were kept in the network if each of the nodes appeared in each other’s top 10 most similar nodes. The input data were searched against annotated reference spectra of the MS/MS library within GNPS. For the visualization of compounds from the dereplication hits, the results were exported and viewed directly with the pie-chart creating tool (nodeCharts plugin for Cytoscape) within Cytoscape 3.7.1.

### Genome Sequencing and *de novo* Assembly Identification of Selected QSI Active Marine Bacterial Strains

Selected marine bacterial strains, isolated from week 3 onward, from the first collection of sponge samples that showed significant QSI activity were subjected to *de novo* assembly identification based on bacterial genomes by 1st Base, Singapore. Bacterial isolates were inoculated into a 250 mL Erlenmeyer flask containing 100 mL of liquid media (Difco^TM^ Marine Broth 2216 supplemented with 0.015 g/L nalidixic acid sodium salt, 0.05 g/L potassium dichromate) and incubated at 25°C with 150 rpm shaking for 14 days. The bacterial cells were pelleted down using centrifuge and sent to 1st Base, Singapore for genomic workup. Briefly, the bacterial genomic DNA was extracted from each cell pellet using the Quick-DNA^TM^ fungal/bacterial microprep kit (Zymo Research) protocol. The quality and quantity of the genomic DNA were measured using PicoGreen, Nanodrop and gel electrophoresis. The samples passed the QC measurement and hence proceed for library preparation using QIAseq FX DNA library kit from QIAGEN. The quality of the libraries was measured using TapeStation 4200, PicoGreen and qPCR. The libraries were then pooled according to the protocol recommended by Illumina and proceeded straight to sequencing using the MiSeq platform at 2 × 251 PE format with QC reads assembled *de novo* using SPAdes 3.11.1 (Bankevich et al., 2012). Paired end Illumina sequences were first removed of sequence adaptors and reads with low quality scores using bbduk of the BBTools Packages^3^. The resulting contigs of >1000 bp were subjected to MEGABLAST against the NCBI Nucleotide database. All genomes were subsequently annotated using the RAST pipeline (Overbeek et al., 2014).

## Results

### Marine Microbes Associated With *Cliona patera*: Culture-Independent Method

A total of 16,381 unique OTUs were identified from the four sample groups, Stem, Inner cup, Outer cup, and Seawater. The Stem group had the highest mean number of unique OTUs (3268 ± 203.2 standard deviation), including 25 bacterial phyla, 66 bacterial classes, five archaeal phyla, and 11 archaeal classes. This was followed by the Inner cup with 3000 (±431.9) unique OTUs (25 bacterial phyla, 62 bacterial classes, six archaeal phyla, and 12 archaeal classes) and Outer cup with 2901 (±349.6) unique OTUs (24 bacterial phyla, 59 bacterial classes, four archaeal phyla, and 12 archaeal classes). Taken together, *C. patera* had an average of 5522 (±613.1) unique OTUs comprising of 26 bacterial phyla, 74 bacterial classes, six archaeal phyla, and 12 archaeal classes. The seawater group had the lowest number of OTUs with 1998 (±233.8), consisting of 26 bacterial phyla, 66 bacterial classes, six archaeal phyla, and 12 archaeal classes (Supplementary Table 2).

**FIGURE 1:**
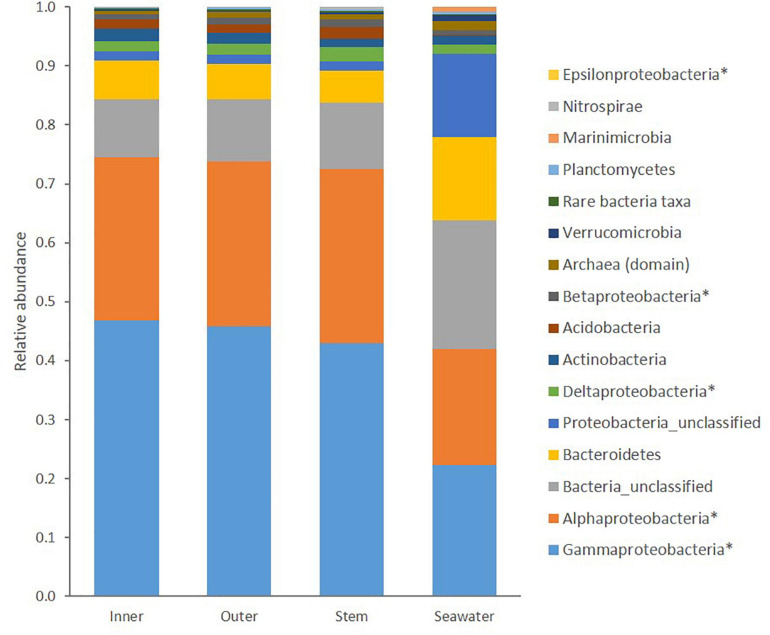
Taxonomic composition of prokaryotic communities in the Inner cup, Outer cup, and Stem of *Cliona patera* and ambient seawater. Asterisks (^∗^) denote classes within the phylum Proteobacteria, and all representatives of the Domain Archaea have been grouped.

Within *C. patera*, *Proteobacteria*, particularly the classes *Gammaproteobacteria* and *Alphaproteobacteria*, dominated the sponge microbiome making up more than 70% of the prokaryotic community (Figure 1). The seawater group was also dominated by more than 40% *Proteobacteria* of the classes *Gammaproteobacteria* and *Alphaproteobacteria*, but also contained a larger proportion of *Bacteroidetes* compared to the sponge samples. Overall, the sponge microbiomes were significantly different from the seawater (PERMANOVA: Pseudo-*F* = 35.249, *P* = 0.001; Supplementary Figure 2).

There were significant differences among some of the sponge and seawater groups for the alpha diversity metrics: Richness (S_*obs*_; *F* = 5.4, *p* = 0.00527), Chao (*F* = 1.911, *p* = 0.154), Inverse Simpson (*F* = 4.45, *p* = 0.0123), and Shannon (*F* = 18.51, *p* < 0.0001). Tukey’s HSD tests revealed differences among all groups – the seawater and sponge (Inner, Outer, and Stem) group, as well as within the sponge when comparing the Inner and Outer cup to the Stem (Table 1). The sponge groups exhibited higher richness (S_*obs*_) and evenness compared to the seawater. Within *C. patera*, the diversity and evenness of the prokaryotic community was significantly lower in the cup as compared to the Stem. The Inner cup and Outer cup had similarly even prokaryotic communities. For both richness metrics, S_*obs*_ and Chao, the Inner cup group had the highest standard deviation in contrast to the other groups which indicated greater variance in the number of OTUs per sample.

**TABLE 1:** Alpha diversity metrics for prokaryotic communities in *Cliona patera* and ambient seawater.

Group	Richness (S_*obs*_)	Richness (Chao)	Evenness (Shannon)	Diversity (inv. Simpson)
Inner	3897.25 ± 1177.21^a^	7999.51 ± 3473.79	0.65 ± 0.02^ac^	26.43 ± 4.60^a^
Outer	3560.13 ± 586.83^b^	7318.48 ± 1986.14	0.65 ± 0.02^bc^	27.61 ± 5.74^b^
Stem	3964.63 ± 383.50^c^	7676.98 ± 1370.09	0.68 ± 0.02^c^	39.96 ± 13.58^ab^
Seawater	2410.80 ± 343.93^abc^	5096.26 ± 692.7	0.60 ± 0.01^abc^	32.29 ± 2.26

*Different letters represent significant pairwise comparisons, where groups with the same letters indicated significant difference between them.*

**FIGURE 2:**
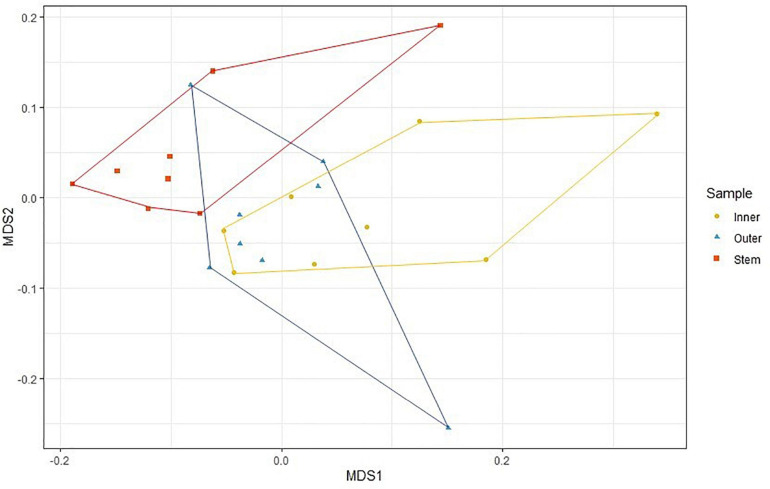
nMDS plot of prokaryotic community structure in the Inner cup, Outer cup, and Stem of *Cliona patera*.

PERMANOVA analysis did not reveal any significant differences between the microbial communities of the six sponges sampled in this study when each sponge was considered as a whole (Pseudo-*F* = 1.1118, *P* = 0.192), or between sponges collected in January and February (Pseudo-*F* = 0.8920; *P* = 0.608). However, within *C. patera*, PERMANOVA analysis revealed significant differences among the regions of the sponge (Pseudo-*F* = 1.6642, *P* = 0.009), specifically between the Inner cup and Stem, as well as the Outer cup and Stem (Table 2). When visualized in the nMDS plot the Inner and Outer cup samples appear grouped more closely than the Stem samples (Figure 2), but there was no differences in dispersion among groups (PERMDISP: *F* = 0.4539, *P* = 0.719).

**TABLE 2:** Pairwise comparisons of prokaryotic community structure (PERMANOVA) in *Cliona patera* Inner cup, Outer cup, and Stem.

*C. patera*	PERMANOVA	
	*t*	*P*
Inner cup, Outer cup	0.927	0.695
Inner cup, Stem	1.551	**0.005**
Outer cup, Stem	1.323	**0.014**

*Bold represent significant pairwise comparisons.*

Generalized linear models supported the PERMANOVA analysis, confirming the distinction between the sponge and seawater prokaryotic communities, as well as between the sponge cup and stem prokaryotic communities. Between the sponge and seawater groups, 98.8% of the 500 OTUs tested contributed significantly (*P* < 0.01) to the dissimilarity, of which 52 OTUs were only present in the sponge group (Supplementary Table 3). The genus *Ruegeria* was not found in the seawater group but was present in all sponge samples. Within the sponge samples, the GLM identified 21.2% of the 500 most abundant OTUs as contributing significantly to the differences detected between the cup and stem (Table 3 and Supplementary Table 4).

**TABLE 3:** Highly abundant (>0.1 mean relative abundance of total community ± SD) OTUs identified as contributing significantly (*P* < 0.01) to the differences between the cup and stem of *Cliona patera*.

OTU	Phylum	Lowest taxonomic classification	Mean relative abundance	
			Cup	Stem
4	*Proteobacteria*	Order *Chromatiales*	1.690 ± 0.226	0.632 ± 0.107
6	*Proteobacteria*	Class *Gammaproteobacteria*	1.715 ± 0.186	0.575 ± 0.266
8	*Proteobacteria*	Order *Rhodobacteraceae*	1.393 ± 0.188	0.453 ± 0.141
15	*Proteobacteria*	Class *Gammaproteobacteria*	1.165 ± 0.339	0.341 ± 0.144
20	*Proteobacteria*	Order *Rhodospirillales*	0.568 ± 0.091	0.422 ± 0.132
27	*Proteobacteria*	Family *Erythrobacteraceae*	0.706 ± 0.195	0.145 ± 0.067
28	*Proteobacteria*	Class *Gammaproteobacteria*	0.683 ± 0.096	0.162 ± 0.081
30	*Proteobacteria*	Class *Betaproteobacteria*	0.401 ± 0.102	0.291 ± 0.045
33	*Proteobacteria*	Order *Rhodospirillales*	0.337 ± 0.068	0.240 ± 0.071
34	*Proteobacteria*	Class *Gammaproteobacteria*	0.304 ± 0.077	0.237 ± 0.067
36	*Proteobacteria*	Class *Gammaproteobacteria*	0.397 ± 0.068	0.141 ± 0.077
37	*Proteobacteria*	Family *Rhodospirillaceae*	0.288 ± 0.051	0.247 ± 0.116
43	*Proteobacteria*	Class *Gammaproteobacteria*	0.328 ± 0.048	0.112 ± 0.029
57	*Proteobacteria*	Class *Alphaproteobacteria*	0.210 ± 0.048	0.153 ± 0.038
61	*Bacteroidetes*	Family *Flavobacteriaceae*	0.252 ± 0.054	0.093 ± 0.022
67	*Actinobacteria*	Genus *Ilumatobacter*	0.181 ± 0.047	0.038 ± 0.020
72	*Proteobacteria*	Genus *Porphyrobacter*	0.244 ± 0.060	0.053 ± 0.013
74	*Proteobacteria*	Class *Gammaproteobacteria*	0.179 ± 0.040	0.126 ± 0.024
89	*Proteobacteria*	Order *Rhodospirillales*	0.125 ± 0.020	0.103 ± 0.032
91	*Bacteroidetes*	Family *Flavobacteriaceae*	0.179 ± 0.071	0.028 ± 0.027
93	*Proteobacteria*	Class *Gammaproteobacteria*	0.109 ± 0.025	0.090 ± 0.034
94	*Proteobacteria*	Order *Myxococcales*	0.082 ± 0.033	0.104 ± 0.068
95	*Proteobacteria*	Class *Gammaproteobacteria*	0.143 ± 0.025	0.052 ± 0.013
98	*Actinobacteria*	Class *Actinobacteria*	0.140 ± 0.036	0.045 ± 0.017
100	*Proteobacteria*	Class *Alphaproteobacteria*	0.104 ± 0.022	0.086 ± 0.027
102	*Nitrospirae*	Genus *Nitrospira*	0.037 ± 0.027	0.149 ± 0.119
103	*Actinobacteria*	Class *Actinobacteria*	0.133 ± 0.033	0.043 ± 0.008
106	*Actinobacteria*	Order *Acidimicrobiales*	0.132 ± 0.031	0.035 ± 0.013
107	*Proteobacteria*	Genus *Haliea*	0.107 ± 0.017	0.074 ± 0.014
110	*Bacteroidetes*	Genus *Robiginitalea*	0.140 ± 0.059	0.024 ± 0.010
113	*Proteobacteria*	Genus *Thiohalomonas*	0.117 ± 0.038	0.025 ± 0.019
135	*Bacteroidetes*	Family *Flavobacteriaceae*	0.106 ± 0.021	0.030 ± 0.006
139	*Proteobacteria*	Family *Rhodospirillaceae*	0.112 ± 0.050	0.022 ± 0.010
147	Unclassified	Bacteria	0.107 ± 0.037	0.018 ± 0.009

### Isolation of Marine Microbes Associated With *Cliona patera*: Culture-Dependent Method

**FIGURE 3:**
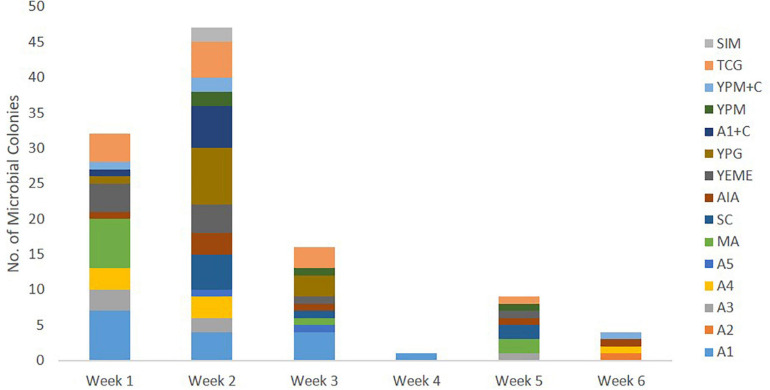
Number of marine bacterial colonies isolated from samples of *Cliona patera*, NP1 and NP6, over a 6-week incubation period in various marine media.

Culture-dependent methods were used to isolate bacteria associated with *C. patera* from different parts of the sponge colonies in order to assess their ability to interfere with bacterial quorum sensing system in the *Pseudomonas aeruginosa* PAO1 *lasB-gfp* biosensor. The first collection of *C. patera* samples, namely from NP1 and NP6, yielded a total of 110 marine bacterial strains isolated over a 6-week incubation period, using 15 different marine culture media (Figure 3). Sixty-four and 46 bacterial strains were isolated from the two sponges, NP1 and NP6, respectively. In addition, the majority of the marine bacterial colonies were isolated from sponge samples obtained from the cup region as compared to the stem, with 85 and 25 bacterial strains, respectively. In addition, colonies that were not considered unique were selected to prevent bias. Of the 15 different marine culture media used for the isolation of bacterial strains, A1, TCG, YPG, MA, and YEME provided the highest yield of isolated colonies, with 16, 13, 12, 11, and 10 strains, respectively (Supplementary Figure 3). The number of isolated bacterial strains peaked at week 2 from 13 marine media (Figure 3). However, the weekly number of new bacterial strains appearing on marine media started to tail off from week 3 onward. For instance, on week 5 and 6, of the 15 different media, only seven (i.e., A3, MA, SC, AIA, YEME, YPM, and TCG) and four (e.g., A2, A4, AIA, and YPM + C) marine media types, respectively, showed the appearance of new bacterial colonies (Figure 3).

### Anti-quorum Sensing Activity of Marine Bacterial Extracts

**FIGURE 4:**
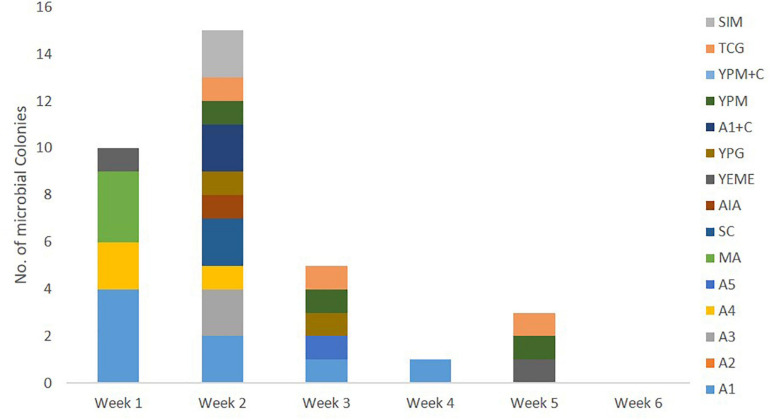
Number of QSI active marine bacterial colonies, with more than 60% florescence inhibition, isolated from samples of *Cliona patera*, NP1 and NP6, over a 6-week incubation period in various marine media.

All 110 marine bacterial strains isolated from samples derived from sponge colonies NP1 and NP6 underwent small-scale fermentation in order to obtain sufficient organic extracts for evaluation of their QSI activity using the biomonitor strain, *Pseudomonas aeruginosa* PAO1 *lasB-gfp*. The organic extracts from cultures of 34 (30.9%) marine bacterial strains exhibited moderate (24 bacterial strains with 60–80% inhibition) to significant (10 bacterial strains with >80% inhibition) florescence reduction on the biosensor PAO1 *lasB-gfp* strain when tested at 100 μg/mL (Figure 4). A majority of the QSI active extracts, about 44% (15 bacterial extracts), were prepared from marine bacterial strains picked solely from week 2 of the incubation period (Figure 4). In addition, the marine media, including A1, A4, MA, TCG, and YPM, gave the highest number of QSI active marine bacterial strains. In spite of the lower number of marine bacterial strains isolated from the stem region of *C. patera*, the proportion of QSI active strains, showing more than 60% florescence inhibition, was comparable to the overall cup region (both inner and outer cup), with 36% and 29.4%, respectively. With the emphasis on slow growing marine bacterial strains, 18 QSI active organic extracts of bacterial strains, isolated from week 3 onward, were further subjected to QSI assay in a dose dependent manner ranging from 1.563 to 100 μg/mL. Twelve crude extracts showed some degree of anti-quorum sensing activity in a dose-dependent manner (Supplementary Figure 4).

### Identification and MS-Based Molecular Networking of Selected QSI Active Marine Bacterial Colonies

*De novo* assembly of bacterial genome for bacterial identification was performed on selected marine bacterial strains with extracts that showed QSI florescence inhibitory activity of more than 55%. The criterion used for the identification was based on genome annotation using RAST. It was revealed that these QSI active bacterial strains belonged to either class *Bacilli* (phylum *Firmicutes*) or class *Alphaproteobacteria* (phylum *Proteobacteria*) (Table 4). The bacterial strains were found to be affiliated with *Labrenzia alba* (strains #91 and #93), *Bacillus stratosphericus* (strain #41), *Ruegeria arenilitoris* (strain #48), and *Staphylococcus haemolyticus* (strain #53) (Table 4).

**TABLE 4:** Overview of best BLAST hits of 16S rRNA gene from genomes of selected colonies of interest showing QSI activity.

Bacterial Strain	Description	Phylum/Class	% Identical sites	% Pairwise identity	% GC	NCBI Accession #	% QS Inhibition
#41	*Bacillus stratosphericus*	*Firmicutes/Bacilli*	99.9%	99.9%	55.2%	MZ328876	58.9%
#48	*Ruegeria arenilitoris*	*Proteobacteria/Alphaproteobacteria*	99.1%	99.1%	55.5%	MZ328874	68.2%
#53	*Staphylococcus haemolyticus*	*Firmicutes/Bacilli*	99.4%	99.4%	51.1%	MZ328872	84.8%
#91	*Labrenzia alba*	*Proteobacteria/Alphaproteobacteria*	98.2%	98.2%	55.9%	MZ328867	78.8%
#93	*Labrenzia alba*	*Proteobacteria/Alphaproteobacteria*	98.2%	98.2%	55.9%	MZ328866	67.5%

**FIGURE 5:**
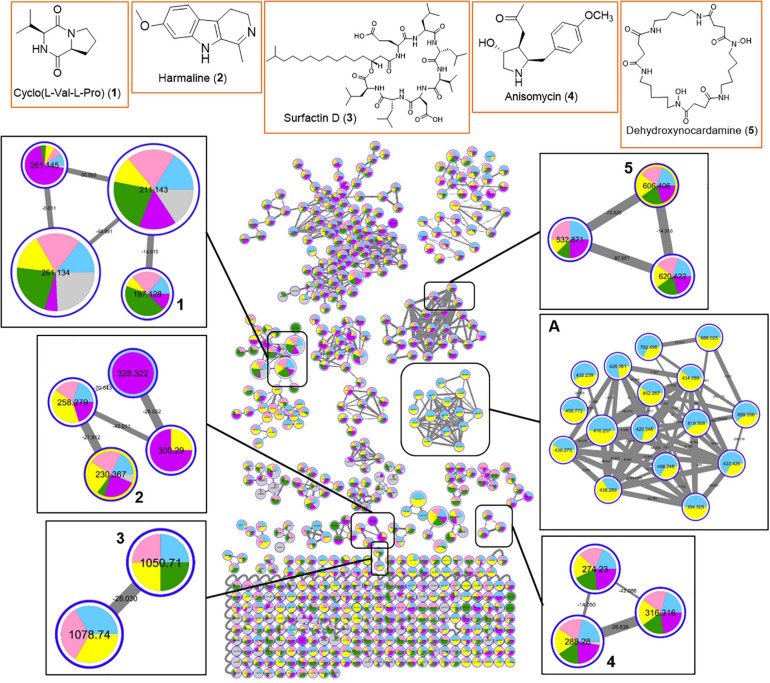
Molecular network of 591 parent ions detected in extracts of five QSI active marine bacterial strains. Blue: strain #53; Pink: strain #91; Yellow: strain #48; Green: strain #93; Purple: strain #41; Gray: MeOH blank. Within each box, the nodes represent the number of ions detected in the bacterial extracts while the node size represents the relative abundance of the different parent ions. Within each node, the relative abundance of a particular ion detected in the different bacterial strains is depicted by the sizes of the colored wedge. The edge thickness reflects the similarity between each parent ions with thicker edge showing higher similarity. Nodes with associated numbers **1** to **5** correspond to the detected compounds and their chemical structures are shown in the top row of boxes.

In order to gain insight into the natural products chemistry of these QSI active bacterial extracts, the mass spectrometry-based metabolomic approach based on molecular networking platform (GNPS) was performed. A total of 591 parent ions (shown as molecular networking clusters in Figure 5) were detected in QSI active extracts derived from five marine bacterial strains, including #41, #48, #53, #91, and #93 (Figure 5). Of the 591 parent ions, compound dereplication based on the GNPS mass spectral library was performed and revealed a total of 120 compound hits. A number of noteworthy natural products, namely diketopiperazines (e.g., compound **1**), harmaline (**2**), surfactin D (**3**), anisomycin (**4**), and dehydroxynocardamine (**5**), were detected in the molecular networking clusters (Figure 5). In addition, a majority of identical/similar ionizable molecules were observed to be present in the extracts of all five marine bacterial strains. For instance, molecular families related to dehydroxynocardamine and anisomycin were found in all bacterial extracts. One molecular networking cluster, containing 16 nodes with *m/z* ranging from 394.505 to 819.508, was unique to strains #53 (class *Bacilli*) and #48 (class *Alphaproteobacteria*) (refer to box A in Figure 5). Moreover, this cluster showed no library hits in the GNPS database. As both marine bacterial strains #91 and #93 are related to *Labrenzia alba*, their molecular signatures were almost identical with only a few ionizable molecules unique to either strains (Supplementary Figure 5).

**FIGURE 6:**
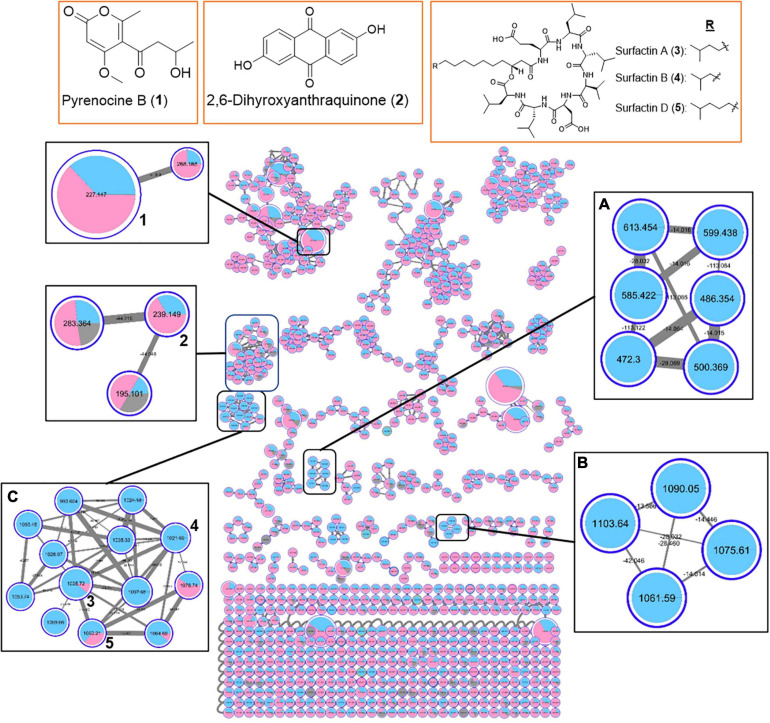
Molecular network of 973 parent ions detected in 11 marine bacterial strains belonging to class *Bacilli* (phylum *Firmicutes*) (Blue) and class *Alphaproteobacteria* (phylum *Proteobacteria*) (Pink). Nodes due to MeOH blank are in gray. Within each box, the different nodes represent the different ions detected in the bacterial extracts while the node sizes represent the relative abundance of the different parent ions. Within each node, the relative abundance of a particular ion detected in the different bacterial strains is depicted by the sizes of the colored wedge. The edge thickness reflects the similarity between each parent ions with thicker edge showing higher similarity. Nodes with associated numbers **1** to **3** correspond to the detected compounds and their chemical structures are shown in the top row of boxes.

The MS-based molecular networking analyses were further expanded to include extracts from 11 marine bacterial strains having different degrees of QSI activity (Supplementary Table 5). From the organic extracts of 11 QSI active bacterial strains, a total of 973 parent ions were detected (Figure 6). Additional new structural classes were also observed, including pyrenocine B, 2,6-dihydroxyanthraquinone and their derivatives (Figure 6). The molecular networkings comparison between two classes of bacteria, belonging to either class *Bacilli* or class *Alphaproteobacteria*, revealed the presence of a number of molecular families that are unique to each bacterial class. For instance, two molecular families, designated in boxes A and B in Figure 6, are only detected in bacterial strains belonging to class *Bacilli*. These two molecular families did not yield any hits from the GNPS library and are potential new molecules. Furthermore, the diversity of surfactin-related molecules, including potential new surfactin derivatives, appeared to be detected predominantly in bacterial strains belonging to class *Bacilli* (box C in Figure 6). Taken together, the molecular networking clusters of QSI active bacterial extracts revealed the present of diverse classes of natural products.

## Discussion

### Microbial Communities Associated With *Cliona patera*: Culture-Independent Method

The Neptune’s Cup sponge, *Cliona patera*, was characterized by a complex and diverse prokaryotic microbiome with more than double the number of distinct OTUs detected in the surrounding seawater. The prokaryotic community structure of the six *C. patera* sponges examined were all similar and distinct from the seawater, suggesting a host-specific microbiome, consistent with previous sponge microbiome research (Schmitt et al., 2007; Sharp et al., 2007; Turon et al., 2018). Moreover, there was no difference in the prokaryotic community structures between the two sponges sampled in January and 1 month later in February. However, additional sampling would be required to assess long-term temporal stability in the microbiomes of *C. patera*.

Interestingly, *C. patera* supported distinct symbiont communities within the cup and stem portions of the sponge. While this is the first study examining the microbiome of *C. patera*, the microbiomes of several *Cliona* species have been studied, namely *Cliona celata*, *Cliona delitrix*, *Cliona lobata*, *Cliona orientalis*, *Cliona thomasi*, *Cliona tumula*, *Cliona varians*, and *Cliona viridis* (Jeong et al., 2015; Thomas et al., 2016; Ramsby et al., 2018; Easson et al., 2020; Meenatchi et al., 2020; Mote et al., 2020; Sacristán-Soriano et al., 2020). This is, however, the first study of a Clionid sponge to identify distinct microbiomes separated into different sections of an individual sponge. The difference in prokaryotic community structure found in the cup and stem of the sponges is likely related to the differences in structural composition of the tough fibrous stem compared to the cup, and the physiological demands on the cup tissue, as it is regularly regrown from turtle predation. Additional research should be conducted to examine the role of the microbiome in sponge function for both the cup and stem of *C. patera*.

Of the OTUs contributing to the differences in microbiomes of the sponge cups and stems, the majority of OTUs (78.3%) were present in higher relative abundance in the cup of *C. patera.* OTU 6 was the most abundant, significant OTU in the cup of *C. patera*, with a relative abundance of 1.715 ± 0.186 in the cup compared to 0.575 ± 0.266 in the stem. OTU 6 was a *Gammaproteobacteria* that matched 99% identity in BLAST to an uncultured bacterium isolated from the coral *Galaxea fascicularis* (Accession KU351051) and 99% identity to an uncultured bacterium retrieved from the Caribbean coral *Montastraea faveolata* (Sunagawa et al., 2009). On the other hand, OTU 4, which belonged to the order *Chromatiales* (also within class *Gammaproteobacteria*), was the most abundant OTU found in the stem of *C. patera* and matched 100% identity in BLAST to an uncultured bacterium isolated from surface seawaters in the East China Sea (Accession KU173719), as well as another uncultured bacterium found in marine sediments in the Philippines (Garren et al., 2009).

Similar to previous studies on the microbiome of marine sponges and within the sponge family Clionaidae, the microbial communities of *C. patera* were predominantly composed of Proteobacteria (relative abundance: 70%); specifically, *Gammaproteobacteria* and *Alphaproteobacteria* (Webster and Thomas, 2016; Pita et al., 2018; Sacristán-Soriano et al., 2020). This high abundance of Proteobacteria support the placement of *C. patera* within the LMA sponges, as predicted by Moitinho-Silva et al. (2017). The other dominant taxa included *Bacteroidetes* (6.05%), *Actinobacteria* (1.82%), *Acidobacteria* (1.66%), the Archaea domain (0.77%), *Planctomycetes* (0.22%), *Verrucomicrobia* (0.2%), *Nitrospirae* (0.2%), *Marinimicrobia* (0.02%), and a large chunk of bacteria remained unclassified (10.5%). Although present in the sponge, the phylum *Chloroflexi* was not a dominant group, unlike reports for a majority of marine sponges (Thomas et al., 2016). In addition, the commonly described exclusively sponge-specific phylum *Poribacteria* was not evident in *C. patera* (Jeong et al., 2015). Only one individual sequence read of *Poribacteria* was detected in one of the seawater samples.

*Cliona patera* did not harbor any *Cyanobacteria*, although it is a bacterial group commonly reported in sponges (Mariani et al., 2001). This absence is consistent with previous research which states that while most phototrophic species are affiliated with *Cyanobacteria*, sponges in the *Clionaidae* family are a notable exception (Schönberg et al., 2006). Instead, the dinoflagellate, *Symbiodinium* has been reported to form symbiotic partnerships with 13 *Clionaidae* species (Schönberg et al., 2005). For instance, vertical transmission of *Symbiodinium* in *Cliona viridis* has been observed (Ramsby et al., 2017) and it appears that the metabolism of *C. viridis* is related to the photosynthetic activities of *Symbiodinium* (Bourne and Webster, 2013). However, the presence of eukaryotes was not evaluated in this study.

The genus *Ruegeria*, phylum *Proteobacteria*, which was present in all sponge samples, was not detected in the seawater group. *Ruegeria* (class *Alphaproteobacteria*) is a genus mainly comprised of marine bacteria and has been repeatedly isolated and cultured from diverse sponge species (Karimi et al., 2019). This genus belongs to the *Roseobacter* clade, which is an ecologically relevant and major marine group equipped with a diverse range of metabolic activities, including the production of novel bioactive compounds (Martens et al., 2007; Luo and Moran, 2014). For instance, a *Ruegeria* strain associated with the sponge *Suberites domuncula* has been reported to produce exocellular cyclic dipeptides (cell signaling compounds) aiding in symbiosis with their hosts (Mitova et al., 2004a,b). Research has also shown that *Ruegeria* species are able to derive sulfur from dimethylsulfoniopropionate, an abundant compound available in marine environments (Wirth et al., 2020). A strain of *Ruegeria* isolated from the marine sponge *Suberites domucula* was also shown to exhibit anti-bacterial activity toward *Bacillus subtilis* (Mitova et al., 2004a,b). *Ruegeria* cultured from Irciniidae sponges showed mild antimicrobial activity against *Staphylococcus aureus* in another study (Esteves et al., 2013). Incidentally, from the culture dependent method used in this study, a *Ruegeria* related bacterial strain #48 was isolated and its crude organic extracts showed quorum sensing inhibitory activity. In addition to unknown molecular families, various classes of known natural products, such as surfactin and anisomycin related molecules were detected in the molecular networking of the bacterial extract (Figure 5).

### Quorum Sensing Inhibitory Activity of Marine Bacteria-Associated With *Cliona patera*: Culture-Dependent Method

Studies have shown that only a fraction of microbial diversity is accurately represented in culture-dependent methods (Connon and Giovannoni, 2002). In order to recover and assess the biomedical potential of marine bacterial strains from sponge samples of *Cliona patera*, a range of marine media, including low nutrient agar, as well as extended incubation period (up to 6 weeks) were used in this study. These methods resulted in the isolation of 110 bacterial colonies from sponge samples. Moreover, the number of media used in the study played an important role in the isolation since no single medium yielded majority of bacterial isolates (Figure 3). These isolation strategies have been used successfully for the cultivation of unique slow-growing bacterial strains with pharmaceutical importance (Olson et al., 2000; Cho and Giovannoni, 2004; Jensen et al., 2005; Gontang et al., 2007; Hameş-Kocabaş and Uzel, 2012; Choi et al., 2015).

A significant number of bacterial extracts, about 30.9%, initially tested at 100 μg/mL showed moderate (60–80%) to significant (>80%) fluorescence reduction in the biosensor PAO1 *lasB-gfp* strain (Figure 4). A majority of the QSI active marine bacterial strains were observed in marine microbes isolated from week 2 of the incubation. This is probably due to the higher number of marine bacterial strains being isolated at that time point. QSI active bacterial strains continued to be detected from bacterial strains isolated after more than 2 weeks of incubation. The percentage of QSI active strains from each sponge colony of NP1 and NP6 was 21.8% and 43.5%, respectively. This relatively high percentage of marine bacteria exhibiting QS inhibitory activity is not unexpected as sponge-associated microbes are known to produce quorum sensing quenching compounds (Saurav et al., 2016; Borges and Simões, 2019; Ong et al., 2019; Singh et al., 2020). This relatively high percentage of QSI active bacterial strains was also detected from both the cup and stem regions of the sponge colonies. To our knowledge, this study is the first of its kind on the occurrence of marine bacterial strains having QS inhibitory activity isolated from sponge samples taken from different parts of the sponge host.

It is not surprising that the QSI active bacterial strains uncovered in this study belonged to either phylum *Firmicutes* (class *Bacilli*) or phylum *Proteobacteria* (class *Alphaproteobacteria*) since these phyla are known to harbor diverse bioactive compounds (Aleti et al., 2015; Timmermans et al., 2017; Buijs et al., 2019). Phylum *Proteobacteria*, in particular, was also one of the major taxonomic bacterial groups revealed from the 16S rRNA amplicon sequencing of the sponge samples. In fact, a recent survey of the literature reported that QS-inhibitory marine bacteria belonged to several classes, including *Alphaproteobacteria* (20.5%), *Gammaproteobacteria* (26.6%), *Actinobacteria* (6.1%), *Bacilli* (37.7%), and *Flavobacteria* (8.8%) (Zhao et al., 2019). Furthermore, a range of QS inhibitors and antimicrobial agents, including diketopiperazines, aromatic polyketides, lipopeptides, furanones and cyclic depsipeptides and peptides, have been reported from marine bacterial strains isolated from a variety of marine samples, such as sediments and marine macroorganisms (Indraningrat et al., 2016; Ma et al., 2018; Borges and Simões, 2019; Chen et al., 2019; Zhao et al., 2019).

### MS/MS Molecular Networking of QSI Active Marine Bacterial Extracts

MS-based molecular networking of organic extracts prepared from selected QSI active marine bacterial strains, belonging to class *Bacilli* and class *Alphaproteobacteria*, revealed diverse chemistry (Figures 5, 6). This metabolomic approach based on MS/MS molecular networking has been used for compound dereplication, as well as for effective screening and detection of potential novel bioactive molecules in extracts derived from natural sources (Yang et al., 2013; Fox Ramos et al., 2019). From a total of 591 precursor ions, derived from initial analysis of five QSI active marine bacterial extracts that showed more than 50% QS inhibitory activity, a range of natural products molecular family clusters, ranging from siderophores (e.g., dehydroxynocardamine), pyrrolidine derivatives (e.g., anisomycin), biosurfactants (e.g., surfactin D), diketopiperazines [e.g., cyclo(L-Val-L-Pro)], and indole alkaloids (e.g., harmaline), were detected (Figure 5). This chemical space is extended to include molecular families of other structural classes, such as aromatic polyketides (e.g., 2,6-dihydroxyanthraquinone) and pyrone-derivatives (e.g., pyrenocine B), as well as addition surfactin-derivatives when the organic extracts of 11 QSI active strains were analyzed (Figure 6). Despite the biotechnological applications and ecological functions, such as biosurfactants, antibiotics and metal chelators, of some of these molecules, their quorum sensing inhibitory activities have not been reported (Stubbendieck et al., 2019; Sajid et al., 2020). The only exception are the diketopiperazine class of compounds where certain members were reported to modulate bacterial quorum sensing system by binding to the receptors of LuxR family (Holden et al., 1999; Abbamondi et al., 2014). Moreover, diketopiperazines display diverse biological properties, including antibacterial, antitumor, antifungal and antiviral activity, making them attractive sources of therapeutic agents (Ortiz and Sansinenea, 2017).

A majority of identical or similar ionized molecules were found to be present in the extracts of all bacterial strains. However, there are specific molecular family clusters or nodes that are restricted to one or two bacterial strains. For instance, molecular clusters containing 16 nodes with *m/z* ions ranging from 394.505 to 819.508 were only detected in marine bacterial strains #53 and #48, related to *Staphylococcus haemolyticus* and *Ruegeria arenilitoris*, respectively (box A in Figure 5). Moreover, ions from this cluster did not show any compound hits in the GNPS database. When the molecular networking clusters were simplified to two taxonomic marine bacterial groups belonging to class *Firmicutes* and class *Alphaproteobacteria*, it was interesting to observe that specific molecular family clusters were only found from the later class (boxes A and B in Figure 6). Certain classes of natural products, such as known and new surfactin analogs, were also found predominately in class *Firmicutes* (box C in Figure 6). Taken together, the molecular networking of QSI active bacterial extracts revealed diverse chemical space with potentially novel bioactive compounds awaiting mining from these marine microbes associated with *Cliona patera*.

## Conclusion

In conclusion, the current study provided insights into the unique microbiome of the Neptune’s Cup sponge, *C. patera*, and sets baseline microbial data for possible future biomonitoring programs in predicting the status of reef environments in Singapore. The sponge hosted 5,222 distinct OTUs, belonging mainly to the phylum *Proteobacteria*, particularly classes *Gammaproteobacteria* and *Alphaproteobacteria*. In addition, a number of these associated marine bacteria, belonging to classes *Bacilli* and *Alphaproteobacteria*, showed promising candidates as sources of novel quorum sensing inhibitory molecules as revealed by their MS-based molecular networking profiles. Due to the limited time, these sponges were only sampled twice within a short period of 1.5 months. Further research should focus on the microbiome stability of *C. patera* over a longer period of time, as well as genes related to microbiome function to advance our understanding of the sponge adaptation and resilience in view of the high sedimentation environment in Singapore’s coastal waters.

## Data Availability Statement

The datasets presented in this study can be found in online repositories. The names of the repository/repositories and accession number(s) can be found in the article/Supplementary Material.

## Author Contributions

LD, LT, XH, and NK conceived the study. LD and XH performed the experiments and acquired the data related to the culture-independent method while LT, NK, and JO were involved in the experiments and data analysis related to the culture-dependent method. JG and MP were involved in acquiring and processing of LC-MS/MS data and molecular networkings. KT and JN were involved in sample collections. All authors revised and accepted the final version of the manuscript.

## Conflict of Interest

The authors declare that the research was conducted in the absence of any commercial or financial relationships that could be construed as a potential conflict of interest.

## Footnotes

1 https://jgi.doe.gov/data-and-tools/bbtools/

2 https://www.mothur.org/wiki/MiSeq_SOP

3 http://gnps.ucsd.edu
