# Supplementary material for: Assessing the Diversity and Biomedical Potential of Microbes Associated With the Neptune’s Cup Sponge, Cliona patera
Source: Front Microbiol. 2021 Jun 29;12:631445. doi: 10.3389/fmicb.2021.631445 (PMC8277423; doi:10.3389/fmicb.2021.631445)
Supplement: Supplementary file 1 [file Data_Sheet_1.docx]

Supplementary Material

**Supplementary Figures**

**
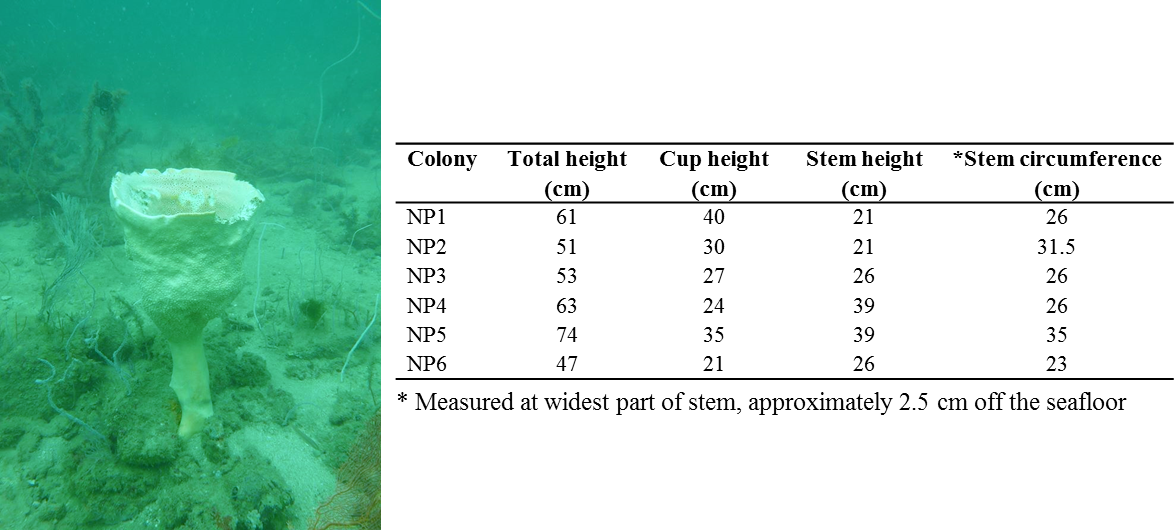
**

**Supplementary Figure S1**. Neptune’s Cup sponge, *Cliona patera*, found on the seabed at the Singapore Strait and dimensions of six sponge colonies (Photo by Dr Karenne Tun).


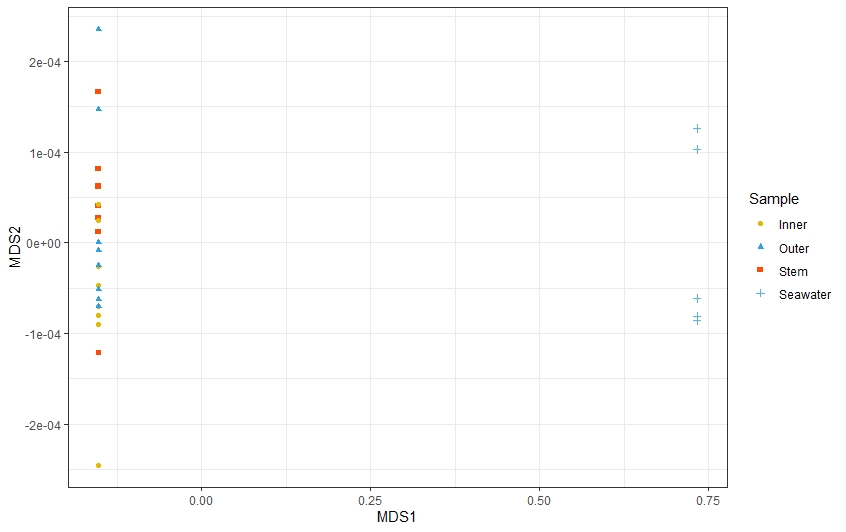


**Supplementary Figure S2**. nMDS plot of prokaryotic community structure in the sponge; *Cliona patera* and ambient seawater based on Bray-Curtis similarity. Stress level = 0.00073809.

**Supplementary Figure S3**. Number of marine microbial colonies isolated from samples of *Cliona patera*, NP1 and NP6, over a 6-week incubation period in various marine media.

**
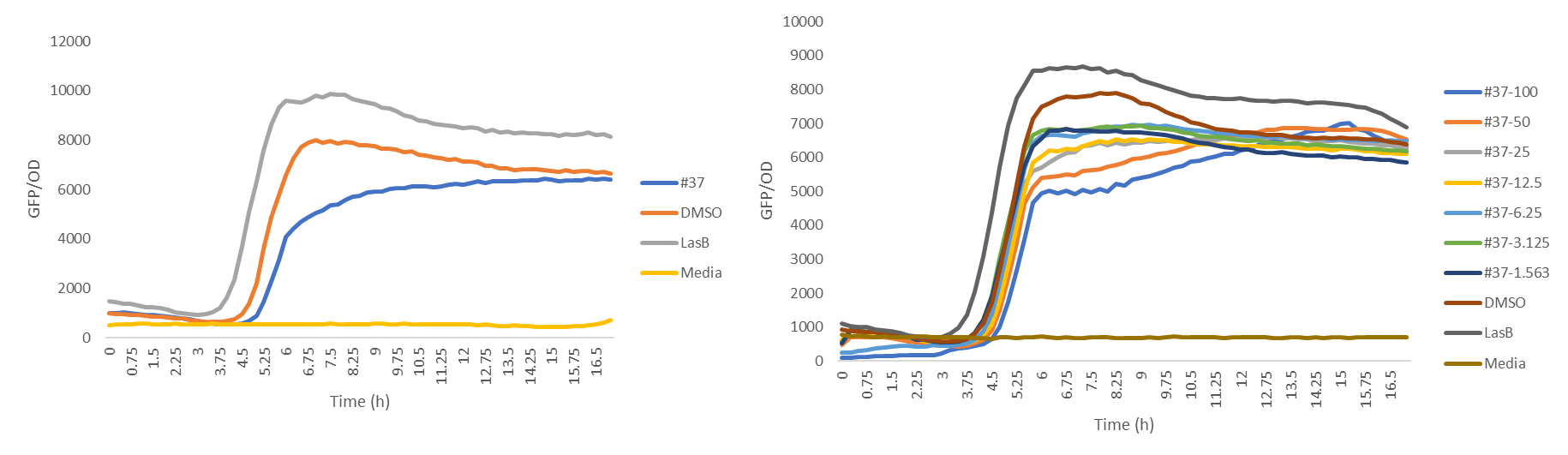
**

**Supplementary Figure S4**. Quorum sensing inhibitory (QSI) activity of extract derived from marine bacterial isolate #37: Typical single-dose tested at 100 μg/mL (left graph) and dose-dependent inhibition curves tested at concentrations of 100 µg/mL to 1.563 µg/mL (right graph). QSI bioassay is based on the *Pseudomonas aeruginosa* PAO1 *lasB-gfp* biosensor strain.

**
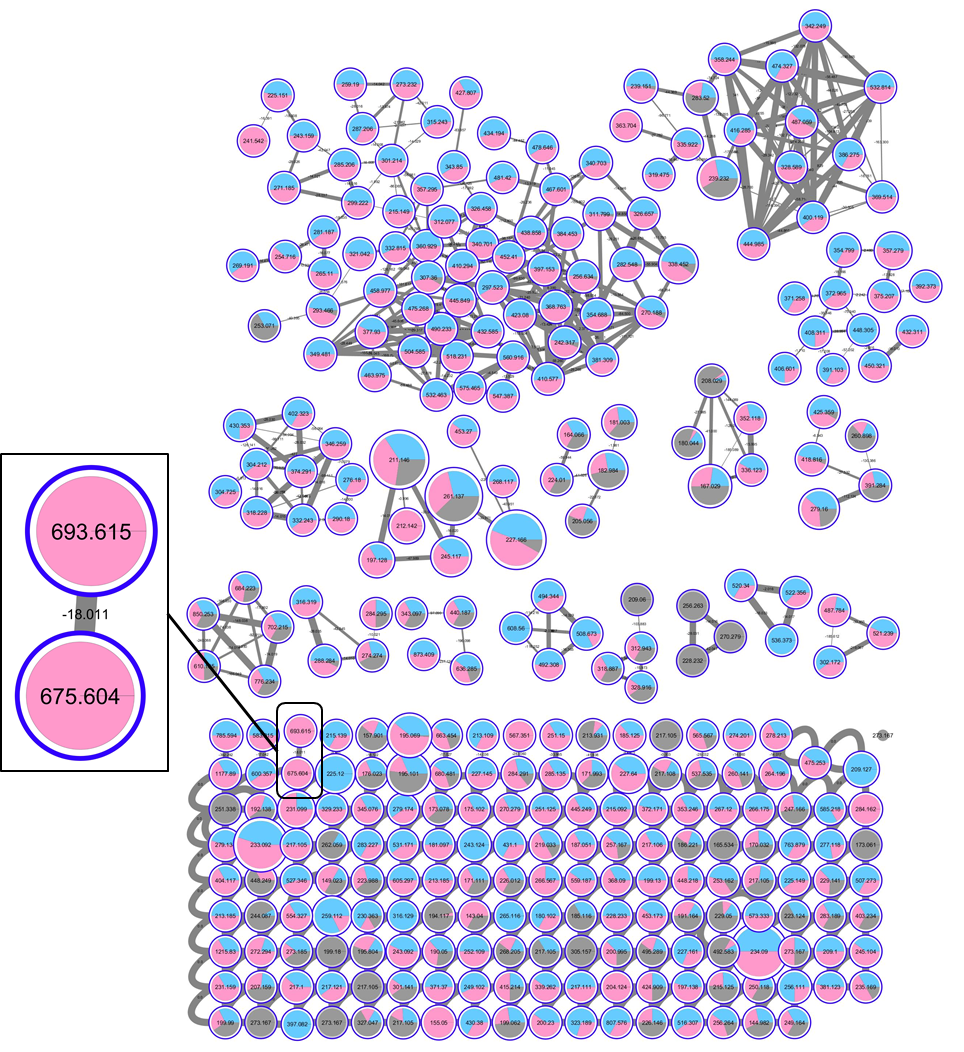
**

**Supplementary Figure S5**. Molecular networks of 323 parent ions detected in the extracts of two *Labrenzia alba* related bacterial strains #91 (blue) and #93 (pink). Grey: MeOH Blank.

**Supplementary Tables**

**Supplementary Table S1.** A summary of media composition, including marine culture media and other media used in the study. All chemicals were purchased from Sigma-Aldrich Chemicals unless otherwise stated.

| **Medium** | **Composition (g/L)** | **Antibiotic Solution** |
| --- | --- | --- |
| A1 | 2g peptone, 10g soluble starch, 4g yeast extract, 18g agar, 1L artificial seawater (ASW) | 0.015g nalidixic acid sodium salt and 0.05g potassium dichromate |
| A2 | 1g dipotassium phosphate, 10g agar, 1g calcium chloride, 0.2g iron chloride, 1g magnesium sulphate, 0.1g manganese sulphate, 1g potassium nitrate, trace element solution (1mL)  Trace element solution: dissolve H_3_BO_3_ (285mg), MnCl_2_.4H_2_O (180mg), ZnCl_2_ (10.5mg), Na_2_MoO_4_.2H_2_O (39mg), CoCl_2_.H_2_O (4mg), CuCl_2_.2H_2_O (4.3mg) in 100ml distilled water | 0.025g cycloheximide and 0.015g nalidixic acid sodium salt |
| A3 | 2g peptone, 5g starch, 2g yeast extract, 22g sea salt, 18g agar, 1L distilled water | 0.015g nalidixic acid sodium salt and 0.05g potassium dichromate |
| A4 | 1g peptone, 5g starch, 2g yeast extract, 18g agar, 500ml ASW, 500ml distilled water | 0.1g cycloheximide and 0.015g nalidixic acid sodium salt |
| A5 | 18g agar, 500ml ASW, 500ml distilled water | 0.1g cycloheximide and 0.015g nalidixic acid sodium salt |
| Marine Agar | 55.1g Marine Agar powder (purchased from Scharlau), 1L distilled water | 0.015g nalidixic acid sodium salt and 0.05g potassium dichromate |
| Starch Casein Agar | 1g casein, 10g starch, 15g agar, 1L ASW | 0.08g cycloheximide and 0.015g nalidixic acid sodium salt |
| Actinomycete Isolation Agar | 22g Actinomycete Isolation Agar, 1L ASW | 0.1g cycloheximide and 0.015g nalidixic acid sodium salt |
| YEME | 4g glucose, 10g malt extract, 4g yeast extract, 18g agar, 1L ASW | 0.015g nalidixic acid sodium salt and 0.05g potassium dichromate |
| YPG | 10g glucose, 5g peptone, 5g yeast extract,  18g agar, 1L ASW | 0.015g nalidixic acid sodium salt and 0.05g potassium dichromate |
| A1+C | 1g calcium carbonate, 2g peptone, 10g soluble starch, 4g yeast extract, 18g agar, 1L ASW | 0.015g nalidixic acid sodium salt and 0.05g potassium dichromate |
| YPM | 2g peptone, 4g mannitol, 2g yeast extract, 18g agar, 1L ASW | 0.015g nalidixic acid sodium salt and 0.05g potassium dichromate |
| YPM+C | 1g calcium carbonate, 2g peptone, 4g mannitol, 2g yeast extract, 18g agar, 1L ASW | 0.015g nalidixic acid sodium salt and 0.05g potassium dichromate |
| TCG | 5g casein, 4g glucose, 3g tryptone, 18g agar, 1L ASW | 0.015g nalidixic acid sodium salt and 0.05g potassium dichromate |
| SIM | 0.1g calcium carbonate, 0.4g casein, 0.2g dipotassium phosphate, 0.1g magnesium sulphate, 0.5g potassium nitrate, 1g starch, 15g agar, 1L ASW | 0.1g cycloheximide and 0.015g nalidixic acid sodium salt |
| MBA | 55.1g Difco™ Marine Agar 2216 powder (purchased from Fisher Scientific), 1L distilled water | NA |
| Marine broth | 37.4g Difco™ Marine Broth 2216 powder, 1L distilled water | NA |
| ABTGC | AB minimal medium supplemented with 7.4 µM thiamine, 0.01 M glucose and 0.01 M casamino acids. AB minimal medium consists of 15.1 mM ammonium sulfate, 33.7 mM sodium phosphate dibasic, 22 mM potassium dihydrogen phosphate, 50 mM sodium chloride, 1 mM magnesium chloride hexahydrate, 100 µM calcium chloride dehydrate and 1 µM iron (III) chloride hexahydrate | NA |

**Supplementary Table S2.** The mean number of OTUs (± standard deviation) found in each sample groups, as well as the total number of bacterial phyla, bacterial classes, archaeal phyla, and archaeal classes for each group. The group *C. petera* represents the combination of the Stem, Inner cup and Outer cup.

|  | **OTUs** | **Bacterial phyla** | **Bacterial classes** | **Archaeal phyla** | **Archaeal classes** |
| --- | --- | --- | --- | --- | --- |
| Stem | 3268 ± 203.2 | 25 | 66 | 5 | 11 |
| Inner cup | 3000 ± 431.9 | 25 | 62 | 6 | 12 |
| Outer cup | 2901 ± 349.6 | 24 | 59 | 4 | 12 |
| *C. patera* | 5522 ± 613.1 | 26 | 74 | 6 | 12 |
| Seawater | 1998 ± 233.8 | 26 | 66 | 6 | 12 |

**Supplementary Table S3.** Identified OTUs absent in the seawater group but present in *C. patera*. These OTUs contributed significantly (P < 0.01) to the differences between *C. patera* and seawater group.

| OTU | Phylum | Lowest Taxonomic Classification |
| --- | --- | --- |
| 38 | *Proteobacteria* | Family *Rhodobacteraceae* |
| 88 | *Proteobacteria* | Family *Rhodobacteraceae* |
| 90 | *Proteobacteria* | Genus *Ruegeria* |
| 102 | *Nitrospirae* | Genus *Nitrospira* |
| 129 | *Proteobacteria* | Genus *Haliea* |
| 142 | *Proteobacteria* | Family *Nitrosomonadaceae* |
| 143 | *Actinobacteria* | Class *Actinobacteria* |
| 150 | *Proteobacteria* | Class *Betaproteobacteria* |
| 163 | *Actinobacteria* | Order *Acidimicrobiales* |
| 180 | Unclassified | Bacteria |
| 185 | *Actinobacteria* | Genus *Ilumatobacter* |
| 203 | *Proteobacteria* | Family *Rhodospirillaceae* |
| 213 | *Proteobacteria* | Family *Rhodospirillaceae* |
| 219 | *Proteobacteria* | Phylum *Proteobacteria* |
| 221 | *Proteobacteria* | Class *Alphaproteobacteria* |
| 234 | *Proteobacteria* | Class *Alphaproteobacteria* |
| 236 | *Proteobacteria* | Family *Rhodobacteraceae* |
| 244 | *Actinobacteria* | Genus *Ilumatobacter* |
| 264 | *Bacteroidetes* | Order *Cytophagales* |
| 286 | Unclassified | Bacteria |
| 292 | *Proteobacteria* | Family *Rhodobacteraceae* |
| 295 | Unclassified | Bacteria |
| 298 | *Proteobacteria* | Order *Rhizobiales* |
| 300 | *Proteobacteria* | Family *Rhodobacteraceae* |
| 302 | *Bacteroidetes* | Family *Flavobacteriaceae* |
| 308 | Unclassified | Bacteria |
| 315 | *Proteobacteria* | Family *Rhodospirillaceae* |
| 327 | *Proteobacteria* | Genus *Rhodovulum* |
| 331 | Unclassified | Bacteria |
| 343 | *Proteobacteria* | Class *Alphaproteobacteria* |
| 370 | Unclassified | Bacteria |
| 372 | *Acidobacteria* | Order *Gp10* |
| 379 | *Proteobacteria* | Class *Deltaproteobacteria* |
| 386 | Unclassified | Bacteria |
| 392 | Unclassified | Bacteria |
| 406 | *Proteobacteria* | Family *Rhodobacteraceae* |
| 416 | *Proteobacteria* | Class *Alphaproteobacteria* |
| 422 | *Bacteroidetes* | Family *Saprospiraceae* |
| 440 | Unclassified | Bacteria |
| 450 | *Proteobacteria* | Class *Alphaproteobacteria* |
| 454 | *Proteobacteria* | Class *Gammaproteobacteria* |
| 456 | *Proteobacteria* | Class *Alphaproteobacteria* |
| 461 | *Acidobacteria* | Phylum *Acidobacteria* |
| 466 | Proteobacteria | Class *Alphaproteobacteria* |
| 472 | Unclassified | Bacteria |
| 478 | Unclassified | Bacteria |
| 486 | *Proteobacteria* | Class *Alphaproteobacteria* |
| 490 | *Proteobacteria* | Class *Gammaproteobacteria* |
| 492 | Unclassified | Bacteria |
| 493 | *Proteobacteria* | Class *Gammaproteobacteria* |
| 495 | *Proteobacteria* | Class *Alphaproteobacteria* |
| 496 | *Proteobacteria* | Family *Rhodospirillaceae* |

**Supplementary Table S4.** Highly abundant (> 0.001 mean relative abundance of total community ± SD) OTUs identified as contributing significantly (P < 0.01) to the differences between the cup and stem of *Cliona patera*.

| OTU | Mean Relative Abundance | | Phylum | Lowest Taxonomic Classification |
| --- | --- | --- | --- | --- |
|  | **Cup** | **Stem** |  |  |
| 4 | 1.690 ± 0.226 | 0.632 ± 0.107 | *Proteobacteria* | Order *Chromatiales* |
| 6 | 1.715 ± 0.186 | 0.575 ± 0.266 | *Proteobacteria* | Class *Gammaproteobacteria* |
| 8 | 1.393 ± 0.188 | 0.453 ± 0.141 | *Proteobacteria* | Order *Rhodobacteraceae* |
| 15 | 1.165 ± 0.339 | 0.341 ± 0.144 | *Proteobacteria* | Class *Gammaproteobacteria* |
| 20 | 0.568 ± 0.091 | 0.422 ± 0.132 | *Proteobacteria* | Order *Rhodospirillales* |
| 27 | 0.706 ± 0.195 | 0.145 ± 0.067 | *Proteobacteria* | Family *Erythrobacteraceae* |
| 28 | 0.683 ± 0.096 | 0.162 ± 0.081 | *Proteobacteria* | Class *Gammaproteobacteria* |
| 30 | 0.401 ± 0.102 | 0.291 ± 0.045 | *Proteobacteria* | Class *Betaproteobacteria* |
| 33 | 0.337 ± 0.068 | 0.240 ± 0.071 | *Proteobacteria* | Order *Rhodospirillales* |
| 34 | 0.304 ± 0.077 | 0.237 ± 0.067 | *Proteobacteria* | Class *Gammaproteobacteria* |
| 36 | 0.397 ± 0.068 | 0.141 ± 0.077 | *Proteobacteria* | Class *Gammaproteobacteria* |
| 37 | 0.288 ± 0.051 | 0.247 ± 0.116 | *Proteobacteria* | Family *Rhodospirillaceae* |
| 43 | 0.328 ± 0.048 | 0.112 ± 0.029 | *Proteobacteria* | Class *Gammaproteobacteria* |
| 57 | 0.210 ± 0.048 | 0.153 ± 0.038 | *Proteobacteria* | Class *Alphaproteobacteria* |
| 61 | 0.252 ± 0.054 | 0.093 ± 0.022 | *Bacteroidetes* | Family *Flavobacteriaceae* |
| 67 | 0.181 ± 0.047 | 0.038 ± 0.020 | *Actinobacteria* | Genus *Ilumatobacter* |
| 72 | 0.244 ± 0.060 | 0.053 ± 0.013 | *Proteobacteria* | Genus *Porphyrobacter* |
| 74 | 0.179 ± 0.040 | 0.126 ± 0.024 | *Proteobacteria* | Class *Gammaproteobacteria* |
| 89 | 0.125 ± 0.020 | 0.103 ± 0.032 | *Proteobacteria* | Order *Rhodospirillales* |
| 91 | 0.179 ± 0.071 | 0.028 ± 0.027 | *Bacteroidetes* | Family *Flavobacteriaceae* |
| 93 | 0.109 ± 0.025 | 0.090 ± 0.034 | *Proteobacteria* | Class *Gammaproteobacteria* |
| 94 | 0.082 ± 0.033 | 0.104 ± 0.068 | *Proteobacteria* | Order *Myxococcales* |
| 95 | 0.143 ± 0.025 | 0.052 ± 0.013 | *Proteobacteria* | Class *Gammaproteobacteria* |
| 98 | 0.140 ± 0.036 | 0.045 ± 0.017 | *Actinobacteria* | Class *Actinobacteria* |
| 100 | 0.104 ± 0.022 | 0.086 ± 0.027 | *Proteobacteria* | Class *Alphaproteobacteria* |
| 102 | 0.037 ± 0.027 | 0.149 ± 0.119 | *Nitrospirae* | Genus *Nitrospira* |
| 103 | 0.133 ± 0.033 | 0.043 ± 0.008 | *Actinobacteria* | Class *Actinobacteria* |
| 106 | 0.132 ± 0.031 | 0.035 ± 0.013 | *Actinobacteria* | Order *Acidimicrobiales* |
| 107 | 0.107 ± 0.017 | 0.074 ± 0.014 | *Proteobacteria* | Genus *Haliea* |
| 110 | 0.140 ± 0.059 | 0.024 ± 0.010 | *Bacteroidetes* | Genus *Robiginitalea* |
| 113 | 0.117 ±0.038 | 0.025 ± 0.019 | *Proteobacteria* | Genus *Thiohalomonas* |
| 116 | 0.086 ± 0.028 | 0.074 ± 0.020 | *Proteobacteria* | Order *Rhodospirillales* |
| 118 | 0.022 ± 0.019 | 0.003 ± 0.003 | *Proteobacteria* | Class *Gammaproteobacteria* |
| 121 | 0.082 ± 0.027 | 0.069 ± 0.009 | *Proteobacteria* | Class *Gammaproteobacteria* |
| 126 | 0.073 ± 0.026 | 0.080 ± 0.017 | *Proteobacteria* | Order *Chromatiales* |
| 135 | 0.106 ± 0.021 | 0.030 ± 0.006 | *Bacteroidetes* | Family *Flavobacteriaceae* |
| 136 | 0.067 ± 0.027 | 0.072 ± 0.020 | *Proteobacteria* | Class *Gammaproteobacteria* |
| 139 | 0.112 ± 0.050 | 0.022 ± 0.010 | *Proteobacteria* | Family *Rhodospirillaceae* |
| 140 | 0.097 ± 0.026 | 0.030 ± 0.008 | *Proteobacteria* | Class *Gammaproteobacteria* |
| 142 | 0.060 ± 0.037 | 0.071 ± 0.042 | *Proteobacteria* | Family *Nitrosomonadaceae* |
| 144 | 0.068 ± 0.021 | 0.060 ± 0.018 | *Proteobacteria* | Class *Alphaproteobacteria* |
| 147 | 0.107 ± 0.037 | 0.018 ± 0.009 | Unclassified | Bacteria |
| 150 | 0.055 ± 0.029 | 0.068 ± 0.044 | Proteobacteria | Class *Betaproteobacteria* |
| 155 | 0.057 ± 0.013 | 0.054 ± 0.022 | Proteobacteria | Class *Gammaproteobacteria* |
| 162 | 0.088 ± 0.020 | 0.019 ± 0.013 | Unclassified | Bacteria |
| 168 | 0.084 ± 0.030 | 0.020 ± 0.015 | Unclassified | Bacteria |
| 170 | 0.080 ± 0.013 | 0.027 ± 0.008 | *Proteobacteria* | Class *Alphaproteobacteria* |
| 173 | 0.080 ± 0.036 | 0.019 ± 0.009 | *Bacteroidetes* | Family *Flavobacteriaceae* |
| 175 | 0.085 ± 0.022 | 0.016 ± 0.009 | *Proteobacteria* | Class *Gammaproteobacteria* |
| 176 | 0.086 ± 0.039 | 0.013 ± 0.016 | *Proteobacteria* | Class *Gammaproteobacteria* |
| 182 | 0.063 ± 0.016 | 0.046 ± 0.014 | *Proteobacteria* | Class *Alphaproteobacteria* |
| 184 | 0.050 ± 0.021 | 0.052 ± 0.016 | *Proteobacteria* | Class *Alphaproteobacteria* |
| 193 | 0.055 ± 0.016 | 0.044 ± 0.014 | *Proteobacteria* | Class *Gammaproteobacteria* |
| 195 | 0.051 ± 0.009 | 0.046 ± 0.013 | *Proteobacteria* | Class *Deltaproteobacteria* |
| 201 | 0.051 ± 0.006 | 0.047 ± 0.016 | *Proteobacteria* | Class *Alphaproteobacteria* |
| 203 | 0.049 ± 0.013 | 0.043 ± 0.016 | *Proteobacteria* | Family *Rhodospirillaceae* |
| 206 | 0.051 ± 0.012 | 0.039 ± 0.012 | *Acidobacteria* | Order *Gp22* |
| 210 | 0.049 ± 0.017 | 0.038 ± 0.009 | *Proteobacteria* | Class *Deltaproteobacteria* |
| 211 | 0.040 ± 0.016 | 0.048 ± 0.025 | *Proteobacteria* | Family *Rhodospirillaceae* |
| 212 | 0.043 ± 0.013 | 0.040 ± 0.014 | Unclassified | Bacteria |
| 216 | 0.044 ± 0.022 | 0.044 ± 0.014 | *Proteobacteria* | Family *Rhodospirillaceae* |
| 217 | 0.035 ± 0.019 | 0.050 ± 0.018 | *Proteobacteria* | Family *Rhodospirillaceae* |
| 235 | 0.032 ± 0.010 | 0.034 ± 0.007 | Unclassified | Bacteria |
| 239 | 0.041 ± 0.011 | 0.031 ± 0.009 | *Proteobacteria* | Class *Alphaproteobacteria* |
| 276 | 0.025 ± 0.006 | 0.034 ± 0.018 | *Proteobacteria* | Order *Rhodospirillales* |
| 277 | 0.029 ± 0.019 | 0.037 ± 0.015 | *Proteobacteria* | Class *Gammaproteobacteria* |
| 283 | 0.019 ± 0.008 | 0.023 ± 0.013 | *Proteobacteria* | Class *Deltaproteobacteria* |
| 288 | 0.035 ± 0.009 | 0.024 ± 0.007 | Unclassified | Bacteria |
| 289 | 0.025 ± 0.007 | 0.030 ± 0.014 | *Proteobacteria* | *Myxococcales* |
| 295 | 0.031 ± 0.008 | 0.024 ± 0.008 | Unclassified | Bacteria |
| 298 | 0.028 ± 0.010 | 0.031 ± 0.014 | *Proteobacteria* | Order *Rhizobiales* |
| 303 | 0.026 ± 0.007 | 0.027 ± 0.007 | *Proteobacteria* | Order *Myxococcales* |
| 305 | 0.025 ± 0.013 | 0.029 ± 0.011 | *Proteobacteria* | Class *Gammaproteobacteria* |
| 310 | 0.029 ± 0.011 | 0.025 ± 0.007 | *Acidobacteria* | Order *Gp9* |
| 311 | 0.026 ± 0.011 | 0.028 ± 0.009 | *Proteobacteria* | Class *Gammaproteobacteria* |
| 315 | 0.029 ± 0.013 | 0.028 ± 0.012 | *Proteobacteria* | Family *Rhodospirillaceae* |
| 322 | 0.029 ± 0.007 | 0.025 ± 0.007 | Unclassified | Bacteria |
| 329 | 0.027 ± 0.012 | 0.024 ± 0.007 | *Proteobacteria* | Class *Alphaproteobacteria* |
| 330 | 0.044 ± 0.011 | 0.008 ± 0.003 | *Proteobacteria* | Class *Gammaproteobacteria* |
| 333 | 0.025 ± 0.007 | 0.025 ± 0.011 | *Proteobacteria* | Order *Rhodospirillales* |
| 337 | 0.023 ± 0.011 | 0.026 ± 0.013 | *Proteobacteria* | Family *Rhodospirillaceae* |
| 345 | 0.020 ± 0.008 | 0.024 ± 0.008 | *Proteobacteria* | Family *Rhodospirillaceae* |
| 349 | 0.024 ± 0.010 | 0.022 ± 0.006 | *Proteobacteria* | Family *Rhodospirillaceae* |
| 350 | 0.024 ± 0.011 | 0.022 ± 0.004 | *Proteobacteria* | Order *Chromatiales* |
| 353 | 0.022 ± 0.006 | 0.024 ± 0.008 | *Proteobacteria* | Class *Deltaproteobacteria* |
| 358 | 0.023 ± 0.009 | 0.023 ± 0.007 | *Proteobacteria* | Class *Gammaproteobacteria* |
| 384 | 0.021 ± 0.006 | 0.021 ± 0.007 | Unclassified | Bacteria |
| 387 | 0.021 ± 0.010 | 0.021 ± 0.009 | *Proteobacteria* | Class *Alphaproteobacteria* |
| 394 | 0.018 ± 0.005 | 0.021 ± 0.011 | *Proteobacteria* | Family *Rhodospirillaceae* |
| 401 | 0.016 ± 0.004 | 0.020 ± 0.012 | *Proteobacteria* | Phylum *Proteobacteria* |
| 411 | 0.028 ± 0.009 | 0.007 ± 0.006 | *Actinobacteria* | Class *Actinobacteria* |
| 418 | 0.025 ±0.007 | 0.006 ± 0.002 | Unclassified | Bacteria |
| 421 | 0.016 ± 0.008 | 0.015 ± 0.006 | *Proteobacteria* | Family *Rhodospirillaceae* |
| 425 | 0.016 ± 0.006 | 0.018 ± 0.011 | Unclassified | Bacteria |
| 431 | 0.017 ± 0.004 | 0.015 ± 0.005 | *Proteobacteria* | Order *Myxococcales* |
| 432 | 0.019 ± 0.008 | 0.015 ± 0.004 | *Proteobacteria* | Class *Alphaproteobacteria* |
| 433 | 0.016 ± 0.006 | 0.018 ±0.008 | Unclassified | Bacteria |
| 435 | 0.015 ± 0.010 | 0.021 ± 0.008 | *Proteobacteria* | Order *Rhodospirillales* |
| 445 | 0.017 ± 0.005 | 0.017 ± 0.010 | *Proteobacteria* | Class *Alphaproteobacteria* |
| 455 | 0.016 ± 0.004 | 0.017 ± 0.006 | *Acidobacteria* | Order *Gp6* |
| 458 | 0.016 ± 0.004 | 0.016 ± 0.006 | Unclassified | Bacteria |
| 462 | 0.009 ± 0.004 | 0.021 ± 0.014 | *Proteobacteria* | Class *Deltaproteobacteria* |
| 464 | 0.018 ± 0.006 | 0.015 ± 0.004 | *Acidobacteria* | Order *Gp6* |
| 469 | 0.015 ± 0.004 | 0.015 ± 0.009 | *Bacteroidetes* | Family *Saprospiraceae* |
| 479 | 0.022 ± 0.005 | 0.005 ± 0.003 | *Proteobacteria* | Family *Halioglobus* |
| 492 | 0.019 ± 0.006 | 0.008 ± 0.004 | *Proteobacteria* | Family *Rhodospirillaceae* |

**Supplementary Table S5.** Overview of best BLAST hits of 16S rRNA gene from genomes of selected marine bacterial colonies showing QSI activity.

| **Strain** | **Description** | **Phylum/Class** | **% Identical Sites** | **% Pairwise Identity** | **% GC** | **NCBI Accession #** | **% QS Inhibition** |
| --- | --- | --- | --- | --- | --- | --- | --- |
| #41 | *Bacillus stratosphericus* | Firmicutes/  Bacilli | 99.9% | 99.9% | 55.2% | MZ328876 | 58.9% |
| #45 | *Labrenzia alba* | Proteobacteria/ Alphaproteobacteria | 98.2% | 98.2% | 55.9% | MZ328875 | 31.2% |
| #48 | *Ruegeria arenilitoris* | Proteobacteria/ Alphaproteobacteria | 99.1% | 99.1% | 55.5% | MZ328874 | 68.2% |
| #49 | *Labrenzia alba* | Proteobacteria/ Alphaproteobacteria | 98.2% | 98.2% | 55.9% | MZ328873 | 44.0% |
| #53 | *Staphylococcus haemolyticus* | Firmicutes/  Bacilli | 99.4% | 99.4% | 51.1% | MZ328872 | 84.8% |
| #81 | *Bacillus stratosphericus* | Firmicutes/  Bacilli | 99.5% | 99.5% | 55.2% | MZ328871 | 40.9% |
| #84 | *Labrenzia alba* | Proteobacteria/ Alphaproteobacteria | 98.2% | 98.2% | 55.9% | MZ328870 | 19.4% |
| #85 | *Labrenzia alba* | Proteobacteria/ Alphaproteobacteria | 98.2% | 98.2% | 55.9% | MZ328869 | 24.5% |
| #86 | *Bacillus licheniformis* | Firmicutes/  Bacilli | 99.5% | 99.5% | 55.4% | MZ328868 | 16.7% |
| #91 | *Labrenzia alba* | Proteobacteria/ Alphaproteobacteria | 98.20% | 98.20% | 55.90% | MZ328867 | 78.82% |
| #93 | *Labrenzia alba* | Proteobacteria/ Alphaproteobacteria | 98.20% | 98.20% | 55.90% | MZ328866 | 67.52% |
